# Attitudes toward suicidal behaviour among professionals at mental health outpatient clinics in Stavropol, Russia and Oslo, Norway

**DOI:** 10.1186/s12888-016-0976-5

**Published:** 2016-07-27

**Authors:** Astrid Berge Norheim, Tine K. Grimholt, Ekaterina Loskutova, Oivind Ekeberg

**Affiliations:** 1 Diakonhjemmet hospital, Postboks 23, Vinderen, Oslo 0319 Norway; 2 Regional Centre of Violence Traumatic stress and suicide Prevention Eastern Norway, RVTS-East, Postboks 4623, Nydalen, Oslo 0405 Norway; 3 Department of Acute Medicine, Oslo University Hospital Ullevål, Pb 4965, Nydalen, Oslo 0424 Norway; 4 Ekaterina Loskutova, ProPsy, Lermontova str. 239/4, ofice 18, Stavropol, 355041 Russia; 5 Division of Mental Health and Addicion, Oslo University Hospital, Box 4956, Nydalen, Oslo 0424 Norway; 6 Department of Behavioral Sciences in Medicine, Institute of Basic Medical Sciences, Faculty of Medicine University of Oslo, Pb 10/2 Blindern, Oslo, N-0316 Norway

**Keywords:** Attitudes, Health professionals, Mental health, Suicide, Suicidal behaviour

## Abstract

**Background:**

Attitudes toward suicidal behaviour can be essential regarding whether patients seek or are offered help. Patients with suicidal behaviour are increasingly treated by mental health outpatient clinics. Our aim was to study attitudes among professionals at outpatient clinics in Stavropol, Russia and Oslo, Norway.

**Methods:**

Three hundred and forty-eight (82 %) professionals anonymously completed a questionnaire about attitudes. Professionals at outpatient clinics in Stavropol (*n* = 119; 94 %) and Oslo (*n* = 229; 77 %) were enrolled in the study. The Understanding Suicidal Patients (USP) scale (11 = positive to 55 = negative) and the Attitudes Towards Suicide Scale (ATTS) (1 = totally disagree, 5 = totally agree) were used. Questions about religious background, perceived competence and experiences of and views on suicidal behaviour and treatment (0 = totally disagree, 4 = totally agree) were examined.

**Results:**

All groups reported positive attitudes, with significant differences between Stavropol and Oslo (USP score, 21.8 vs 18.7; *p* < 0.001). Professionals from Stavropol vs. Oslo reported significantly less experience with suicidal patients, courses in suicide prevention (15 % vs 79 %) guidelines in suicidal prevention (23 % vs 90 %), interest for suicide prevention (2.0 vs 2.7; *p* < 0.001), and agreed more with the ATTS factors: avoidance of communication on suicide (3.1 vs 2.3; *p* < 0.001), suicide is acceptable (2.9 vs 2.6; *p* = 002), suicide is understandable (2.9 vs 2.7; *p* = 0.012) and (to a lesser extent) suicide can be prevented (4.2 vs 4.5; *p* < 0.001). In both cities, psychiatric disorders (3.4) were considered as the most important cause of suicide. Use of alcohol (2.2 vs 2.8; *p* < 0.001) was considered less important in Stavropol. Psychotherapy was considered significant more important in Stavropol than Oslo (3.6 vs 3.4; *p* = 0.001).

**Conclusions:**

Professionals reported positive attitudes towards helping suicidal patients, with significant differences between cities. A need for further education was reported in both cities, but education was less integrated in mental health care in Stavropol than it was in Oslo. In both cities, psychiatric disorders were considered the major reasons for suicide, and psychotherapy was the most important treatment measure.

## Background

Suicide is a serious health problem and the second leading cause of death in 15–29 year olds worldwide [1, 2]. Health professionals are expected to prevent suicide, but their lack of knowledge and experience with suicidal behaviour has contributed to the perception of these patients as being challenging [3]. Studies have reported that health professionals have felt incompetent and have avoided communication with suicidal patients [3]. Patients have felt vulnerable regarding their rejection by clinical staff, which was most pronounced in somatic care [4]. This may contribute to the increase of suicide risk [5].

In most cultures, mental health professionals are the frontline health professionals who help patients with suicidal behaviour. Cultural, religious and professional backgrounds, as well as knowledge and experience of suicidal behaviour can influence attitudes [6, 7]. The prioritization of suicide prevention is more likely to be neglected in countries with high suicide rates than it is in countries with low rates [8]. Attitudes might be a serious obstacle to the prevention of a problem as widespread as suicide. Neglect might be related to prestige in medical science [9].

Awareness of own attitudes and its impact on decision-making and treatment contribute to understand the patients situation better and improve treatment, education and strategies to prevent suicide as well as cross cultural cooperation. Our study is built on knowledge about risk factors for suicide [10–14], and effective treatment [15–17], all though suicidology is complex and causes might differ within, and between cultures like Norway and Russia [18–20]. Cross-cultural knowledge of attitudes among mental health professional’s are limited, although understanding, competence and attitudes can be essential to whether the patients seek or obtain help [7], and essential to improve suicidal prevention.

Most studies of health professional’s attitudes toward suicidal patients are carried out in somatic hospitals [3, 4]. Comparative studies of mental health professionals working clinical in outpatients clinics have to our knowledge not been performed, all though the number of patients with suicidal behaviour increasingly are treated in outpatient clinics. Considering the serious health problem that suicidal behaviour represents worldwide the existing knowledge concerning this matter from research is sparse [1, 2]. In Russia, were suicide rates have been among the highest in the world, scientific studies about suicide are rare, and cooperation with other health professionals has historically been limited.

In this study, we wanted to explore the attitudes toward suicide among professionals at mental health outpatient clinics in Stavropol, Russia and Oslo, Norway.

### Stavropol, Russia

Stavropol is a city located in the North Caucasus in southern Russia, with a population of about 400 000 inhabitants. The Russian Orthodox Church, Islam and Buddhism are important in the area. Suicide is considered as a sin in most religions, especially in the Russian Orthodox Church and Islam. Religiosity, psychodynamic understanding and collaboration with professionals from other countries were difficult in Soviet times.

Similar to the rest of Russia, Stavropol has experienced great changes from the Soviet era to date. In all of Russia, the suicide rates fell in the first perestroika period, but increased again to peak at 42 per 100 000 per year in 1994. The suicide rates have slowly decreased subsequently, to 23.5 in all of Russia in 2010 (http://www.who.int/mental_health/evidence/atlas/profiles/rus_mh_profile.pdf?ua=1). The suicide rates in the northern and eastern parts of Russia are among the highest in the world, especially among men and indigenous people [21]. The lowest suicide rates in Russia are found in Muslim central Asiatic and western parts of Russia and in the North Caucasus, which reflects the large diversity in the cultural, economic and political situations of the area [22].

In 1995, the Russian Ministry of Public Health issued an order toward the organization of suicidology centres in large cities; however, suicide prevention remains limited because Russia still lacks financial support for a national program for suicidal prevention with a local focus [23].

### Oslo, Norway

Oslo is the capital of Norway and has a population of about 600 000 inhabitants. The Protestant Church, which was a state religion in Norway until 2012, considers suicide as a sin, but is less judgmental than the Russian Orthodox Church and Islam. Its basic attitude is to help suicidal patients and support relatives after suicide [24].

Suicide rates increased in Norway during the last part of the 19th century, to peak in 1988 at 16.8 per 100 000 per year, decreasing to 10.8 in Norway and 11.4 in Oslo in 2008–2012, representing 1.3 % of all deaths [25]. The suicide rates differ within Norway, with higher suicide rates recorded in the north.

As a result of the increase in suicide rates, Norway created a national program for suicide prevention in 1993, as well as first national plan in 1994. National Centre and Regional Centres for suicide research and prevention in the four health regions of Norway were established. In 2008, national guidelines for suicide prevention in mental health care were released and implemented [26], and a new national plan for suicide prevention was established in 2014 [27].

### Differences in mental health care

In Russia, mental health care services underwent large changes after the Soviet era. Traditionally, most psychiatric treatments were carried out in large hospitals with biological orientation and little focus on psychotherapy. Physicians and psychiatrists were responsible for deciding on the treatment, whereas nurses and unskilled professionals had practical, but not formal, responsibility. Psychologists were barely involved in in-patient treatment and currently have no formal responsibility.

During recent decades, Russia has been modernizing its health care, with the creation of additional outpatient clinics. The education of psychologists has increased since 1990 and many psychologists currently work at state psychological centre practice or outpatient clinics, which are connected to mental health hospitals.

Norway has also gone through large changes in mental health care when in-patient clinics were scaled back during recent decades. Nurses and other professional groups were trained and were partly delegated more responsibility for the treatment of these patients via teamwork. From 2001, psychologists were given formal responsibility to consider coercion [28]. A greater number of patients with a moderate risk of suicide were treated in outpatient clinics, which may be challenging for professionals.

### Aims

The present study aimed to identify possible differences in attitudes between the cultures by exploring responses to the following questions: 1) What are the attitudes towards suicidal patients among mental health professionals in Stavropol, Russia and Oslo, Norway? 2) Do attitudes differ according to professional background, gender, age and religion? 3) Are there differences in experiences with suicidal patients, self-assessed competence and understanding of suicidal behaviour according to workplace or profession?

## Methods

### Subjects

This study was based on anonymous responses to paper questionnaires that were collected from professionals working at mental health outpatient clinics in Stavropol and Oslo. Three hundred and forty-eight professionals (82 %) answered the questionnaire: 119 (94 %) from Stavropol and 229 (77 %) from Oslo.

The Understanding Suicidal Patients (USP) scale [29] was used to measure willingness to provide care to suicide attempters and to assess the professional’s understanding and sympathy for these patients. The scale consists of 11 items scored on a five-point Likert scale. Responses were recorded on a scale from 1 (completely agree) to 5 (completely disagree). Some questions were reversed. A sum score was calculated, ranging from 11 (positive) to 55 (negative). Earlier studies have considered scores below 23 as being positive [30, 31]. The reliability measure of the USP scale in the original study was a Cronbach’s alpha of 0.74 [30]. In this study, the Cronbach’s alpha was 0.57.

The Attitudes Towards Suicide Scale (ATTS) [32] was used to measure a broad dimension of attitudes towards suicide, such as suicide as a right, comprehensibility, communication, preventability and taboo. The scale consists of 37 items scored on a five-point Likert scale (1 = totally disagree, 5 = totally agree). The dimensions are found by factor analyses with varimax rotation and Kaiser Normalization. The factor model was generated based on eigenvalues, scree plot and factor loadings.

We added questions about perceived competence, religious background and experiences and views of suicidal behaviour and treatment (0 = totally disagree, 4 = totally agree). The questionnaire was developed to cover cultural differences. Age was recorded by 10-year intervals, from 1 (under 30 years) to 5 (over 60 years), to preserve anonymity.

In Stavropol, the questionnaires were collected from health professionals working with children, adolescents and adults who were also expected to work with suicidal patients in conditions as similar to those observed in the outpatient clinics in Oslo as possible. As the outpatient clinics were less developed, only 10 % of the questionnaires were collected from child and adolescent clinics. Our Russian contributor, EL, asked the health professionals to participate. Forty-two per cent of the questionnaires were collected from professionals working in private outpatient clinics or alone, 32 % from psychiatric hospitals and 26 % from a psychotherapeutic conference, where a few professionals from other parts of Russia completed the questionnaire. The inclusion period was from January to March 2011. The questionnaires were unnamed and analysed in Norway.

In Oslo, the questionnaires were collected from all four health regions, which cover different socio-economic statuses, to obtain a general picture of the Oslo outpatient clinics. Forty-seven per cent of the questionnaires were collected from Child and Adolescent Psychiatry and 53 % from District Psychiatric Centres in Oslo. Department heads were asked to facilitate the study by distributing the questionnaires via internal mail and during staff meetings, and encouraging staff members to participate. The inclusion period was from November 2010 to February 2011. The paper questionnaires were completed anonymously and then labelled with the name of the workplace and a number.

### Statistics

Data are presented as means with 95 % confidence intervals (CIs). The chi-squared test was used for comparisons of categorical data. Student’s *t*-test and analyses of variance were used to compare continuous data between groups. Cronbach’s alpha was used to investigate reliability. For ATTS analysis, factor analyses with varimax rotation and Kaiser Normalization were used to investigate the scale dimensions and Cronbach’s alpha was investigated for each factor. The level of significance was set at *P* < 0.05.

The data were analysed using SPSS software (v. 19–21; SPSS, Chicago, IL and IBM, Armonk, NY).

### Ethics

We contacted The Norwegian Regional Etic Committee of Health South East to clarify whether we needed permission to perform the study. The ethics committee declared that since the study did not involve patients, it was sufficient to obtain permission from the Oslo University Hospitals Privacy Protection that has strict routines for treatment of data. The study is completed according to advice from Oslo University Hospital Privacy Protection.

The Local Stavropol State Medical University Ethics Committee declares that the study does not contradict their principles of operation.

## Results

### Characteristic of the professionals

#### Profession, gender, age and religion

Table 1 display the characteristic of the professionals. Psychologists (52 %) represented the largest group of professionals. The smaller groups, i.e., nurses (16 %) and physicians (14 %), answered similarly; and were merged to improve power. The majority of professionals in Stavropol and Oslo were specialists: nurses (71 % vs 94 %), physicians (69 % vs 45 %) and psychologists (56 % vs 33 %, respectively). As “others” (mostly educators in child and adolescent care) and social workers were unevenly distributed, they were mostly excluded. Seventy per cent of the professionals were women. Professionals were significantly younger in Stavropol than in Oslo. Significantly more professionals from Stavropol compared with Oslo reported a Christian background, mostly from the Russian Orthodox Church in Stavropol and the Protestant Church in Oslo.

**Table 1:** Characteristics of professions in Stavropol and Oslo

	Stavropol	Oslo	Total	*p* value
	*n* = 119 (%)	*n* = 229 (%)	*n* = 348 (%)	
Psychologists	75 (64)	106 (46)	181 (52)	
Nurses	21 (18)	34 (15)	55 (16)	
Physicians	16 (14)	33 (14)	49 (14)	
Others	5 (4)	56 (24)	61 (18)	<0.001
Male	25 (21)	79 (35)	104 (30)	
Female	93 (79)	147 (65)	240 (70)	0.008
Age				
<30 years	42 (36)	16 (7)	58 (17)	
31–40 years	41 (35)	78 (34)	119 (35)	
41–50 years	25 (21)	51 (23)	76 (22)	
>50 years	9 (8)	81 (36)	90 (26)	<0.001
Christians	107 (91)	137 (60)	244 (71)	
Orthodox	87 (74)	1 (0)	88 (26)	
Protestant	0 (0)	121 (53)	121 (35)	
Catholic	18 (15)	9 (4)	27 (8)	
Other Christians	2 (2)	6 (3)	8 (2)	
Other religions	2 (2)	6 (3)	8 (2)	
No religion	8 (7)	85 (37)	93 (27)	<0.001

### Understanding Suicidal Patients scale

Professionals in both cities reported positive attitudes; however, they were significantly less positive in Stavropol than in Oslo (21.8 vs 18.7; *p* < 0.001), single items referred represent the main difference. Nurses reported significantly more positive attitudes than did psychologists in Stavropol (19.5 vs 22.7; *p* = 0.002), but not in Oslo. Gender differences were not statistically significant (Table 2).

**Table 2:** Attitudes towards suicide according to the Understanding Suicidal Patients (USP) scale

	Stavropol	Oslo	*P*
	*n* = 119	*n* = 229	
	Mean (95 % CI)	Mean (95 % CI)	
Scale (11 = positive to 55 = negative)	21.8 (20.9–22.6)	18.7 (18.1–19.2)	<0.001
Men	22.6 (20.5–24.7)	18.7 (17.9–19.5)	<0.001
Women	21.7 (20.7–21.6)	18.6 (17.9–19.3)	<0.001
Psychologists	22.7 (21.7–23.7)	18.4 (17.6–19.2)	<0.001
Physicians	20.5 (17.1–23.9)	18.9 (17.3–20.4)	0.261
Nurses	19.5 (17.6–21.4)	18.0 (16.7–19.3)	0.160
USP items			
1. Patients who have attempted suicide are usually treated well at my workplace	1.7 (1.6–1.8)	1.4 (1.3–1.7)	<0.001
3. I am usually sympathetic and understanding toward a patient that has attempted suicide	1.9 (1.7–2.1)	1.5 (1.4–1.6)	<0.001
4. I do my best for a patient who has attempted suicide, to make them feel safe and cared	1.6 (1.5–1.8)	1.3 (1.2–1.4)	<0.001
5. It is usually difficult to meet a patient who has tried to take his/her life	2.3 (2.1–2.6)	3.0 (2.8–3.2)	<0.001
6. I do my best to speak with a patient who has attempted suicide about his/her personal problems	1.7 (1.5–1.8)	1.3 (1.2–1.4)	<0.001
9. Because patients who have attempted suicide have emotional problems, they need the best possible treatment	1.5 (1.4–1.7)	1.3 (1.2–1.4)	0.006

Scale:

1 = Totally agree, 2 = Partly agree, 3 = Nor agree or disagree, 4 = Partly disagree, 5 = Totally disagree

### Factors according to Attitudes Towards Suicide Scale scores

The factor analysis of ATTS scores initially suggested a 10-factor solution that explained 61 % of the variance. However, the scree plot, eigenvalues and factor loadings showed a four-factor structure that explained 41 % of the variance.

#### Factor one: “Avoidance of communication”

Factor one showed a nine-item structure with items 4, 7, 11, 12, 13, 19, 23, 27 and 33, which explained 19 % of the variance. The factor loadings were between 0.67 and 0.59, and the Cronbach’s alpha was 0.82. More professionals from Stavropol compared with Oslo agreed with this statement (3.1 vs 2.3) (Table 3).

**Table 3:** Attitudes Towards Suicide Scale (ATTS): factors and single items

	Stavropol *n* = 112 Mean (95 % CI)			Oslo *n* = 173 Mean (95 % CI)			Stavropol Oslo *n* = 285	
	Physicians and Nurses	Psychologists	*p*	Physicians and nurses	Psychologists	*p*		*P*
Factor 1: Avoidance of communication	3.4 (3.2–3.6)	3.0 (2.8–3.1)	<0.001	2.3 (2.2–2.4)	2.2 (2.2–2.3)	ns	3.1–2.3	<0.001
Factor 2: Suicide is acceptable	2.8 (2.5–3.1)	2.9 (2.8–3.1)	ns	2.3 (2.2–2.5)	2.8 (2.7–3.0)	<0.001	2.9–2.6	0.002
Factor 3: Suicide is common and understandable	2.7 (2.3–3.0)	2.9 (2.8–3.1)	ns	2.5 (2.4–2.7)	2.8 (2.7–2.9)	0.002	2.9–2.7	0.012
Factor 4: Suicide can be prevented	4.3 (4.1–4.5)	4.1 (4.0–4.2)	ns	4.6 (4.5–4.7)	4.5 (4.4–4.6)	ns	4.2–4.5	<0.001
ATTS single items								
2. Suicide can never be justified	4.0 (3.7–4.4)	3.4 (3.3–3.7)	0.008	3.2 (2.9–3.4)	2.9 (2.7–3.1)	0.024	3.7–3.0	<0.001
3. Suicide is the worst thing to do	3.9 (3.5–4.2)	3.2 (2.9–3.4)	0.003	3.4 (3.1–3.6)	2.9 (2.7–3.1)	0.006	3.4–3.1	0.018
9. Suicide prevention is a duty	4.7 (4.5–4.9)	4.3 (4.1–4.5)	0.017	4.8 (4.6–4.9)	4.5 (4.4–4.6)	0.007	4.4–4.6	0.032
13. Suicide should not be talked about	3.0 (2.5–3.5)	2.4 (2.2–2.6)	0.004	1.6 (1.5–1.8)	1.4 (1.3–1.5)	0.039	2.6–1.5	<0.001
27. I do not understand why people take their life	3.3 (2.9–3.7)	2.5 (2.3–2.7)	<0.001	2.1 (2.0–2.4)	1.8 (1.6–1.9)	0.001	2.8–1.9	<0.001
35. Most suicide is trigged by conflicts	3.1 (2.8–3.1)	3.5 (3.4–3.7)	0.005	2.7 (2.5–2.9)	3.0 (2.9–3.2)	0.003	3.4–2.9	<0.001

Scale:

Do not vote at all = 1, Do not vote = 2, In doubt, depends on = 3, Votes largely = 4, Votes entirely = 5

CI = Confidence Interval

Ns = Non significant

#### Factor two: “Suicide is acceptable”

Factor two showed a seven-item structure with items 5, 16, 20, 29, 32, 34 and 36. This factor explained 12 % of the variance. The factor loadings were between 0.82 and 0.57 and the Cronbach’s alpha was 0.84. Oslo physicians and nurses agreed least with this statement (Table 3).

#### Factor three: “Suicide is common”

Factor three showed a four-item structure with items 14, 15, 25 and 31. This factor explained 6 % of the variance. The factor loadings were between 0.69 and 0.51 and the Cronbach’s alpha was 0.60. Few physicians or nurses agreed with this statement in both cities (Table 3).

#### Factor four: “Suicide can be prevented”

Factor four showed a three-item structure with items 9, 30 and 37. This factor explained 4 % of the variance. The factor loadings were between 0.70 and 0.56 and the Cronbach’s alpha was 0.55. The professionals agreed to this statement, but professionals from Stavropol agreed significant less than the professionals from Oslo (4.2 vs 4.5) (Table 3).

#### Single items from the Attitudes Towards Suicide Scale

Physicians and nurses in both cities and all of the professionals from Stavropol agreed most with the statements: “Suicide can never be justified”, “Suicide is the worst thing to do”, “Suicide should not be talked about” and “I do not understand why people take their life”. Physicians and nurses also agreed more with the statement “Suicide prevention is a duty”, although fewer professionals from Stavropol agreed with this statement. Psychologists in both cities agreed more with the statement, “Most suicides are trigged by conflicts” and in general, professionals from Stavropol agreed most with this statement, (Table 3).

### Experience, competence and interest in suicide prevention; religion and view of suicide

Few professionals in Stavropol reported suicide attempts and deliberate self-harm in their own patients. They also participated in fewer courses (15 % vs 78 %) and had fewer local guidelines in suicide prevention (23 % vs 90 %). In Oslo, significantly more physicians and nurses (23 %) lost patients to suicide than did psychologists (11 %), with no significant differences observed between the cities. Professionals in both cities reported an equal need for further education in suicidology. Twenty-nine per cent of the professionals in Stavropol and 3 per cent in Oslo reported that their religious background determined their view of suicide (Table 4).

**Table 4:** Experience, competence, interest in suicide prevention and view of religion (%)

	Stavropol			Oslo			Stavropol Oslo		
	*n* = 112			*n* = 173			*n* = 285		
	Physicians and nurses %	Psychologists %	*p*	Physicians and nurses %	Psychologists %	*p*	%	%	*p*
Experienced lost of own patient to suicide	23	11	ns	36	14	<0.001	15	23	ns
Experienced suicide attempt in own patient	65	45	ns	79	73	ns	50	76	<0.001
Experience self-harm in own patient	69	58	ns	95	91	ns	62	92	<0.001
Course in suicide prevention	11	19	ns	78	78	ns	15	78	<0.001
Written guidelines; suicide prevention	44	13	*p* = 0.002	87	92	ns	23	90	<0.001
Need education in suicide prevention	70	82	ns	70	73	ns	78	73	ns
My religious background determines my views of suicide	33	27	ns	18	2	*p* = 0.036	29	3	<0.001

Scale: Yes or no

Scale for “need education in suicide prevention” and “religious background”: In very high degree, In fairly high degree, In moderate degree, In limited extent, Not at all. Here % of very high and fairly high degree

Ns = Non significant

### Profession and reported need for further competence

Psychologists in Stavropol agreed least with the statements: “I think my current competence gives me skills and capacity to safeguard a person after an attempted suicide” and “The treatment system works well for persons that have attempted suicide”. They agreed most with the statement: “I need further education to work with patients after attempted suicide.” Stavropol physicians and nurses reported the least interest in suicide prevention, whereas their colleagues in Oslo reported the most interest in this issue. In both Stavropol and Oslo, physicians and nurses gained increased supervision towards suicidal patients compared with psychologists (Table 5).

**Table 5:** Training and competence according to profession and city

	Stavropol			Oslo			Total	
	*n* = 112 Mean (95 % CI)			*n* = 173 Mean (95 % CI)			*n* = 285	
	Physicians and Nurses	Psychologists	*p*	Physicians and Nurses	Psychologists	*p*	Stavropol vs Oslo	*p*
I think my present training has provided me with adequate skills to take care of people who have tried to commit suicide	3.6 (3.2–4.0)	2.4 (2.1–2.7)	<0.001	4.0 (3.8–4.2)	4.0 (3.8–4.1)	ns	2.8–4.0	0.001
I am in need of further training to work with patients who have tried to commit suicide	4.1 (3.8–4.5)	4.6 (4.4–4.7)	0.028	3.8 (3.6–4.1)	3.6 (3.4–3.8)	ns	4.4–3.7	<0.001
Treatment service in mental health care works well for people who have tried to commit suicide	3.6 (3.1-4.0)	2.4 (2.2-2.7)	<0.001	3.6 (3.4-3.7)	3.4 (3.2-3.6)	ns	2.8-3.5	<0.001
Degree of interest in suicide prevention	2.8 (2.4–3.1)	3.2 (2.9–3.5)	0.055	3.9 (3.7–4.1)	3.1 (2.9–3.3)	0.025	3.1–3.8	<0.001
Gained supervision	2.8 (2.5–3.1)	2.4 (2.2–2.6)	0.025	3.5 (3.4–3.7)	3.1 (2.9–3.3)	0.001	2.6– 3.2	<0.001

1 = Totally disagree, 2 = Partly disagree, 3 = Nor agree or disagree, 4 = Partly agree, 5 = Totally agree

CI = Confidence Interval

Ns = Non significant

### Views on causes of suicide, treatment and available treatment

#### Statements on causes of suicide

All professionals agreed that “psychiatric disorder” was the most important reason for suicide. However, “inner turmoil and stress” and “conflicts in the family” were considered as being more important among psychologists in both cities. Professionals from Stavropol agreed significantly less with the statement that “use of alcohol” can cause suicide. Stavropol physicians and nurses agreed significant more than psychologists that suicide is caused by biological changes in the brain (2, 1 vs 1, 6) (Table 6).

**Table 6:** View on suicide issues and treatment

	Stavropol			Oslo			Stavropol Oslo	
	Total *N* = 119 (*n* = 112)			Total *N* = 229 (*n* = 173)			Total *N* = 348 (*n* = 285)	
	Mean (95 % CI)			Mean (95 % CI)			Mean	
	Physicians and Nurses	Psychologists	*p*	Physicians and Nurses	Psychologists	*p*		*p*
Causes of suicide								
Psychiatric disorder	3.4 (3.1–3.6)	3.3 (3.1–3.4)	ns	3.4 (3.3–3.7)	3.3 (3.2–3.4)	ns	3.3–3.4	ns
Inner turmoil and stress	2.5 (2.1–2.9)	3.1 (2.8–3.3)	0.012	2.6 (2.4–2.8)	3.0 (2.8–3.2)	0.003	2.9–2.8	ns
Problems in the family	2.4 (2.1–2.7)	3.0 (2.8–3.2)	0.001	2.7 (2.5–2.9)	2.8 (2.7–3.0)	ns	2.8–2.7	ns
Use of alcohol	2.4 (1.9–2.8)	2.2 (2.0–2.4)	ns	2.8 (2.7–3.0)	2.8 (2.6–2.9)	ns	2.2 –2.8	<0.001
Biological changes in the brain	2.1 (1.7–2.5)	1.6 (1.3–1.8)	0.021	1.9 (1.7–2.1)	1.8 (1.6–2.0)	ns	1.9–1.8	ns
Importance of treatment								
Psychotherapy	3.5 (3.3 –3.8)	3.7 (3.6–3.8)	ns	3.1 (3.0–3.3)	3.6 (3.4–3.7)	<0.001	3.6 –3.4	0.001
Sleep and rest	2.3 (1.9–2.6)	2.1 (1.9–2.3)	ns	3.0 (2.8–3.2)	2.9 (2.7–3.0)	ns	2.2–2.9	<0.001
Psychiatric in-patient treatment	3.0 (2.6–3.3)	2.1 (1.9–2.3)	<0.001	2.8 (2.6–3.0)	2.7 (2.5–2.8)	ns	2.3–2.7	<0.001
Use of medication	3.1 (2.8–3.5)	1.9 (1.7–2.1)	<0.001	2.7 (2.5–2.9)	2.5 (2.3–2.6)	0.050	2.3–2.6	0.002
Family therapy	3.1 (2.8–3.3)	3.3 (3.1–3.4)	ns	2.6 (2.5–2.8)	2.6 (2.4–2.8)	n	3.2–2.6	<0.001
Talk with priest/imam or others in the church	2.7 (2.4–3.0)	2.4 (2.2–2.6)	ns	2.2 (2.0–2.4)	2.0 (1.8–2.2)	ns	2.5–2.1	<0.001
Electroconvulsive therapy	0.7 (0.4–0.9)	0.2 (0.1–0.2)	<0.001	1.4 (1.2–1–6)	1.4 (1.2–1.6)	ns	0.3–1.4	<0.001
Satisfaction with treatment								
Long enough/adequate follow-up	2.9 (2.6–3.2)	2.3 (2.1–2.5)	0.003	2.4 (2.2–2.7)	2.0 (1.8–2.2)	0.003	2.5–2.1	<0.001
Opportunity for hospitalisation, if needed	3.0 (2.5–3.4)	2.4 (2.1–2.6)	0.006	2.8 (2.6–3.0)	2.4 (2.2–2.5)	0.002	2.6–2.4	ns
Follow–up as good as that provided to patients with heart disease	3.1 (2.7–3.5)	2.3 (2.0–2.6)	0.001	2.2 (1.9–2.4)	1.7 (1.5–1.9)	0.013	2.5–1.7	<0.001
The suicide of a patient is a professional failure	2.1 (1.8–2.5)	1.7 (1.5–2.0)	ns	1.4 (1.2–1.7)	1.4 (1.2–1.5)	ns	1.9–1.4	<0.001

0 = Not at all agree, 1 = In limited degree agree, 2 = In moderate degree agree, 3 = In high degree agree, 4 = In high degree agree

CI = Confidence Interval, Ns = Non significant

#### Statements about treatment

All professionals reported a need for further education in suicidology. All professionals, especially the psychologists, considered psychotherapy as an important treatment method, whereas sleep and rest were considered as being less important. Fewer Stavropol psychologists than Stavropol physicians and nurses agreed that “in-patient treatment” and “use of medication” are important factors, and that “family therapy” is important. In Oslo, there were no significant differences between the groups. Stavropol professionals agreed more with the statement that “talking with a priest/imam” is important compared with the Oslo professionals. Electroconvulsive therapy (ECT) was consider with less relevance for treatment in Stavropol than Oslo (0.3 vs 1.4) (Table 6).

#### Statements about the quality of treatment of patients with suicidal behaviour

Physicians and nurses in Stavropol agreed more than did Oslo professionals that suicidal patients get “Long enough/adequate follow-up” and “Opportunity for hospitalization if needed” and are offered “Follow-up similar to that offered to patients with heart disease”. Stavropol professionals agreed more to “suicide of a patient is a professional failure” than the professionals from Oslo (1.9 vs 1.4) (Table 6).

## Discussion

All professionals reported positive attitudes towards patients with suicidal behaviour, with somewhat more positive attitudes observed in Oslo compared with Stavropol. There were differences between the groups regarding whether communication about suicide should be avoided and whether suicide is acceptable, common and understandable or can be prevented. The attitudes seemed to be most influenced by background, such as city and profession. As most professionals in Stavropol reported a Russian Orthodox background, it was not possible to measure the influence of religion, although professionals in Stavropol agreed significant more than professionals from Oslo that religious background influenced their view if suicide. However, the religious influence in Oslo has been described previously, where professionals that reported Christian background agreed significant less to the factor “suicide is acceptable” [33].

In this study, 70 % of the professionals were women, with only minor differences in attitudes detected among genders. Age differences might be a minor bias in questions related to items such as experience. We found no differences in experience of suicide in own patients between the cities; however, Stavropol professionals reported significantly less experience with suicidal behaviour and self-harm, had less education and guidelines and were less interested in suicide prevention. All professionals agreed most with the statements that psychiatric disorders are the most important reason for suicide, and that psychotherapy is the most important treatment.

The USP scale has been used previously in somatic care, where professionals often report less preparedness, knowledge and willingness to help suicidal patients compared with the findings of this study [29–31]. In our study the professionals in Stavropol agreed more than professionals from Oslo that “It is usually difficult to meet a patient who has tried to take his/her life”. A review of previous studies clarifies that insecurity obstacles treatment of patients with suicidal behaviour [3]. A lower degree of supervision, courses, guidelines and interest in suicide prevention in Stavropol might have contributed to less attention to and awareness of the problem, and less positive attitudes in the USP scale. The professionals who had gained most supervision about treatment of suicidal patients reported the most positive attitudes [33]. A previous study from Norway found most positive attitudes among psychiatrists compared to internists and general practitioners [34].

In both cities, nurses showed the most positive attitudes, although this was only significant toward the psychologists in Stavropol. A possible explanation for this finding is that nurses with experience from inpatient clinics have a greater experience in dealing with patients with suicidal behaviour for many hours per day, which might give insight into the patient’s situation and influence attitudes. Psychologists in Stavropol reported the least experience, courses and satisfaction with their own competence, which may decrease their preparedness to work with these patients. They also reported the greatest need and interest in further education as many also express willingness to help. They were least satisfied with the available treatments for mental health problems.

In a study of attitudes among politicians where ATTS was used, suggest the presence of less interest and belief in suicide prevention in countries with high suicide rates compared with countries with low suicide rates [8]. This is also in line with our findings of a higher level of agreement with “Suicide is common and understandable” in Stavropol, and a lower level of agreement with “Suicide can be prevented”.

Professionals in Stavropol were more likely to avoid communication about suicidal ideation than professionals from Oslo, as indicate less awareness of the importance of communication of suicidal ideation. In Norway guidelines recommend screening for suicidal risk factors in all patients who are referred to mental health treatment. Further investigation are required if risk factors are detected [26]. It is essential to address any suicidal ideation in treatment to get help. Stigma and shame among patients and professionals can hinder such communication [2–4, 35].

Occurrence of suicidal ideation, behaviour and reasons for suicide might differ between the cities and within Russia and Norway. Findings from the Northern Russia and Northern Norway show less reported suicidal ideation among patients and professionals in Northern Russia, especial among men, despite the higher suicidal rates, particular in men. Failure to acknowledge and communicate suicidal ideation is suspected to be a risk factor for suicide [18, 20]. Stigmatic attitudes and little awareness of suicidal ideation might hinder patients to get help to be aware needed changes to get a better life. Screening might contribute to more tailored treatments.

Professionals in Stavropol and psychologists in Oslo agreed more to “suicide is acceptable” than the physicians and nurses in Oslo. High acceptance toward suicide is also found in Moscow [36]. Lower suicide rates are found in societies in where suicide is unacceptable, such as strict religious societies [1, 24]; however, stigma and taboo might contribute to the under-reporting of suicide and suicidal behaviour. Conversely, in the Netherlands, where physician-assisted suicide and euthanasia are legal and represent 2.8 % of all deaths, suicide, assisted suicide and euthanasia together represent about threefold the suicide rates observed in Oslo and Stavropol (http://www.who.int/mental_health/evidence/atlas/profiles/rus_mh_profile.pdf?ua=1) [25, 37].

In this study, Oslo physicians and nurses agreed most with “suicide prevention as a duty”, as this might represent a strong connection to the Hippocratic Oath to preserve life and relieve pain. However, physicians and nurses in both cities also reported a higher degree of condemning attitudes towards suicide, which were most pronounced in Stavropol. Condemning attitudes can increase hopelessness, loneliness, despair and shame, which can decrease help seeking and increase suicide risk [35]. A study from northern Russia found cohesion between condemning attitudes in men and own experience of suicidal behaviour, which was interpreted as a risk factor for suicide. This was not observed in Norway or Sweden [20].

Psychiatric disorders were considered the most important causes of suicide in both cities in line with previous studies that highlights mental illness as main reason for suicide [10]. But suicides have many causes, and a study from Northern Russia and Northern Norway found that suicide were less associated with severe mental illness in Russia than Norway [18]. The World Health Organization has stated that mental disorder and harmful use of alcohol contribute to many suicides worldwide [1], also described in several studies [38–40]. In our study professionals from Stavropol emphasised significant less that alcohol can cause suicide, all though the alcohol consume, especially among men are expected to be higher [39, 40]. A more common problem might represent a habitual situation were a coping strategy is neglecting the problem, like found cross- cultural among politicians attitudes toward suicide [8].

Also factors like physical health, [11], psychological coping strategies and sociological and economically factors [5, 10, 12], religiosity [24], culture [6], and lifestyle [13], can influence risk for suicide. In our study the psychologists agreed most to that inner turmoil and stress can cause suicide. They also agreed most to that family problem could cause suicide, but this was only significant in Stavropol. The professional’s only moderate or less agreed that biological changes in the brain could cause suicide.

The complexity of suicide makes it difficult to measure effective treatments, and treatment needed depends on the patient’s condition. Psychotherapy was reported as the most important treatment in both cities, most clearly in Stavropol and among psychologists. A recent register study demonstrated that socio-psychological treatment was effective [15]. The physicians/nurses agreed more than psychologist that inpatient clinics were important, only significant in Stavropol. Physicians/nurses also agreed most that medication was important, highly significant in Stavropol. All though well proven medication like Clozapine [16] and Lithium [17] have significantly decreased suicidal risk among patients with affective disorders, studies of suicidal patients that survived intoxication, highlights that medication also can be a risk factor [41] and cautious use of pharmacotherapy is specially recommended among young patients [14].

Professionals in Stavropol valued family therapy and talking with a priest/imam higher than did the professionals in Oslo, whereas sleep and rest were valued higher in Oslo. Professionals from Stavropol answered close to “not at all agree” to Electro convulsing therapy as treatment, when professionals from Oslo scored somewhat higher. The therapy has effect on major depression, highly associated with suicide. Different practice in application and execution might influence the view [42].

Regarding satisfaction with treatment, professionals in Stavropol were the most satisfied with the follow-up and available treatment, despite our experience of a lower availability of treatments for children and adolescents, and higher mortality in Stavropol compared with Oslo (http://www.who.int/mental_health/evidence/atlas/profiles/rus_mh_profile.pdf?ua=1) [25].

Previous studies from Oslo have found significant elevated risk for suicide and early death after intoxication [43] but insufficient possibilities for treatment and a longer follow-up [44], also is observed in other countries [3, 4, 45, 46]. Neglect of the patients’ problems might lead to insufficient referral to mental health care, where professionals are more positive towards providing help. The professionals agreed only moderately that the patients with suicidal behaviour receive the same quality of treatment as provided to patients with heart disease; and the prestige hierarchy in medicine might influence this [9].

Working with suicidal patients entails an elevated risk for losing patients to suicide, despite the implementation of precautions. This is challenging for professionals. Loss of own patients to suicide often awakens feelings of guilt, sadness and incompetence, which are more difficult to handle if the patient is young and the professionals are still in training, new at the workplace, or are unprepared for suicide and receive little support from colleagues [47].

Professionals in Stavropol agreed significant more than did their colleagues in Oslo that suicide in own patients are a failure of care. High degree of responsibility for the patients might lead to self-condemning attitudes after a patient’s suicide. The burden might lead to stress, feeling incompetent in work and create need for avoidant of suicidal patients to protect oneself. Support and supervision can be crucial to evolve in profession and is highly recommended when working with suicidal patients.

### Strengths and limitations

In this study, we used two different questionnaires that have been used internationally in different cultures and populations to cover different dimensions of attitudes. Together with other questions, the responses to these questionnaires have provided a broad impression of the professional’s attitudes, which was strength of the study.

We found that the two first factors of ATTS were satisfactory, whereas the two next factors had low Cronbach’s alpha. We chose to include them, because they support earlier findings and can have implications for clinical practise. For USP the Cronbach’s alpha was rather low, but our findings were supported by the findings on the separate items. We present the USP sumscore to enable comparisons with other studies using the USP scale.

The sample from Oslo is quite representative for psychiatric outpatient units. In the sample from Stavropol, there were more participants who worked in private practice and who were motivated to attend a conference in psychotherapy. Thus, there is possible that the participants in Stavropol had a more psychotherapeutic attitude than most psychiatrists and psychologists in Russia. Accordingly, they may consider psychotherapy to be more important and use of medication (and ECT) less important than the average Russian professionals.

## Conclusions

Professionals showed positive attitudes according to willingness and understanding, with minor differences observed between Stavropol and Oslo. Stavropol professionals reported less training and experience with suicidal patients compared with their Oslo colleagues. There were significantly different views regarding whether suicide should be discussed, is acceptable and understandable or can be prevented.

Psychiatric disorders were considered the main cause of suicide, and psychotherapy was considered the most important treatment in both cities all though common understanding between professionals was less integrated in Stavropol than Oslo. Our study indicated more positive attitudes in mental health than found earlier in somatic care, where high-risk patients often feel neglected. This clarifies the need for improving co-operation with somatic care, to detect and give qualified treatment to high-risk patients. Support for professionals who lose patients to suicide is recommended.
